# “When the Worlds Change and We Get Old”: Indigenous Older Adults Reflecting on Dementia and Aging

**DOI:** 10.3390/ijerph23060720

**Published:** 2026-05-28

**Authors:** Tamar Ginossar, Erika L. Partridge, John C. Adair, Donica M. Ghahate, Michele M. Quam, Jillian L. Prestopnik, Janice A. Knoefel, Sephira G. Ryman, Gary A. Rosenberg, Vallabh O. Shah

**Affiliations:** 1 Communication and Journalism, University of New Mexico, Albuquerque, NM 87131, USA; 2 Center for Memory & Aging, University of New Mexico Health Science Center, Albuquerque, NM 87106, USA; epartridge@salud.unm.edu (E.L.P.); jadair@salud.unm.edu (J.C.A.); mmquam@salud.unm.edu (M.M.Q.); jprestopnik@salud.unm.edu (J.L.P.); jknoefel@salud.unm.edu (J.A.K.); sryman@mrn.org (S.G.R.); grosenberg@salud.unm.edu (G.A.R.); 3 Department of Neurology, University of New Mexico Health Science Center, Albuquerque, NM 87131, USA; 4 Pueblo of Zuni, Zuni Pueblo, NM 87327, USA; donica.ghahate@ashiwi.org; 5 Mind Research Network, Albuquerque, NM 87106, USA; 6 Department of Internal Medicine, University of New Mexico Health Sciences Center, Albuquerque, NM 87131, USA

**Keywords:** Indigenous health, dementia, Alzheimer’s disease and related dementias, aging, Southwester Pueblos, Native American older adults, caregiving, culturally grounded dementia care, community-engaged research, COVID-19 and social isolation

## Abstract

**Highlights:**

**Public health relevance—How does this work relate to a public health issue?**

- Dementia and aging-related care are major public health concerns, yet Southwestern Pueblo communities remain underrepresented in research on older adults’ needs, experiences, and access to culturally appropriate services.
- The study connects dementia care with broader public health challenges affecting older adults in this community, including caregiving burden, limited specialized dementia resources, and the lasting impacts of the COVID-19 pandemic.

**Public health significance—Why is this work of significance to public health?**

- This work documents Zuni older adults’ perspectives on dementia, aging, caregiving, and service needs through a community-engaged research process approved by the Zuni Tribal Government and co-led by community members.
- The findings show that culturally grounded dementia care must include prevention, screening, caregiver support, education, and specialized healthcare services that reflect community values and lived experiences.

**Public health implications—What are the key implications or messages for practitioners, policy makers and/or researchers in public health?**

- Practitioners and policymakers should support dementia-related services that are both culturally grounded and clinically specialized, including resources for family caregivers and accessible information about dementia prevention, screening, and care.
- Public health planning should address the long-term effects of the COVID-19 pandemic by rebuilding social services, strengthening community gatherings, and reducing loneliness and social isolation among older adults.

**Abstract:**

Background. Understanding the perspectives of Indigenous older adults about aging and dementia in different Indigenous communities can inform the provision of culturally grounded services and advance equity. However, the experiences and perspectives of Southwestern Pueblo communities are under-researched. Methods. Approved by the Zuni Tribal Government and IRB, this community-engaged, qualitative research explored Zuni older adults’ perceptions, experiences, and support needs. Two co-authors who are community members co-led four focus groups and transcribed them. Results. Twenty participants in four focus groups shared holistic perceptions of dementia. Whereas they often described dementia as a part of aging, they also expressed a strong desire for specialized dementia care and information. They expressed a strong commitment to caregiving as a cultural value, along with challenges and the need for resources to support caregiving as part of specialized dementia healthcare. The challenges experienced by community members were exacerbated by the devastating impact of the COVID-19 pandemic, including the loss of loved ones, long-term COVID-19, disruption of gatherings, loneliness and social isolation. Conclusions. The need for culturally grounded services is inseparable from Zuni participants’ requests for specialized dementia services (including prevention, screening, and caregiving). As the first study to report on the impact of the COVID-19 pandemic on Zuni aging, it highlights the importance of re-building social services and gatherings in planning of service provision.

## 1. Literature Review

Indigenous communities in the U.S. and globally experience significant Alzheimer’s disease and Alzheimer’s disease-related dementias (AD/ADRD) disparities. An increasing body of literature documents the importance and complexity of AD/ADRD in Indigenous populations [1,2,3,4,5,6,7,8,9,10], including increased risk [10,11] and limited access to specialized care services [12,13]. Advancing brain health equity requires going beyond the biomedical model. Understanding contributors to cognitive resilience, that may serve as a buffer for the negative impacts of colonization and health inequities [8]. Along with growing evidence on the prevalence and risks related to AD/ADRD in Indigenous populations [14], researchers have noted gaps in the literature [15,16]. Barriers to such research include the rurality of communities, their smaller size, and the increased resources that are needed for community engagement to bridge years of research misconduct and institutional marginalization.

Given the cultural richness and diversity of different Indigenous communities, it is important to avoid their homogenization and presumptions that can lead to “one size fits all” approach to treating dementia in different Indigenous communities. Zuni, a Western New Mexico (NM) Pueblo (Village), is one of 23 recognized Tribes in the State of New Mexico (NM). For thousands of years, its people have upheld a unique language (Shiwi), spiritual beliefs, and religious and cultural systems that cannot be fully shared with outsiders [17,18]. Along with cultural richness, creativity, and resilience, as enduring legacy of colonization, the community has endured historical trauma, economic marginalization, and limited access to healthcare. These are linked with increased community and individual stress and adverse health outcomes, including hypertension, diabetes, and related renal diseases [19]. These morbidities have been shown to increase dementia risk [20,21]. Another contributing factor to inequities in dementia outcomes is the reduction in potential caregivers due to migration out of Indian country [13]. Consequently, Indigenous older adults have are more likely to experience earlier hospital and nursing home admissions compared to other populations [22]. This lack of a well-developed continuum of care for older Indigenous adults diminishes their quality of life and increases mortality [13]. To advance cognitive health equity, it is therefore important to learn about community members’ perceptions of dementia-related continuum of care, and their interest in addressing and treating dementia.

Barriers to dementia care-seeking in the general population include seeing dementia as a normal consequence of aging; attributing its etiology to spiritual, psychological, physical health, or social causes; conceptualizing caregiving as a personal or family responsibility; shame; stigma; the belief that nothing could be done; and negative healthcare experiences. However, more recent exploration of Indigenous perceptions of aging and dementia in Canadian Indigenous communities revealed how such beliefs can have a more complex, dialectical role that can also have positive impact on models of care. Whereas some studies examined Indigenous perceptions of dementia in diverse communities [4,7,8], this is the first study to engage the Zuni Pueblo in such examination. Given the importance of contextual factors in the lived experiences of people with dementia [23], and the cultural variability and richness within different Indigenous communities, research is needed to better understand the experiences of people in different Indigenous communities [12]. To our knowledge, such research has not previously been done with Southwestern Indigenous communities. Given the importance of Indigenous Ways of Knowing, it is important to listen to the community. Understanding the perceptions and experiences of dementia, caregiving, and healthcare access is essential for designing effective strategies that are acceptable to the community [1,24].

This study is part of a long-term collaboration between Zuni community, AD/ADRD healthcare providers and researchers. The overall goal of this stage of the study was to inform this collaboration and healthcare services between dementia clinicians and researchers and the community by identifying the community’s needs for dementia services and information. Using the data for secondary analysis, we sought to answer the following research question: What are participants’ aging- and dementia-related perceptions and experiences?

## 2. Methods

Methodology. This study was guided by a community-engaged qualitative methodology designed to center Pueblo older adults’ own meanings, experiences, and ways of understanding aging and dementia. Rather than applying predetermined categories or restrictive eligibility criteria, the research approach prioritized cultural responsiveness, relational trust, and participant-led sensemaking. Focus groups were selected because they are culturally congruent and well suited for eliciting shared experiences, community perspectives, and points of agreement or difference among participants, particularly in research with marginalized populations [25].

Cultural grounding was central to the methodology. Community-member coauthors who are also healthcare workers led the focus groups and transcription process, helping ensure that the research was conducted in a culturally appropriate manner and that participants’ expressions, including narratives in Shiwi, were preserved. This approach positioned community knowledge as central to the research process rather than as supplemental context. The team’s prior experience conducting focus groups in diverse New Mexico communities, including this Pueblo, further supported the use of this culturally responsive approach [26,27].

Consistent with best practices for working with Indigenous communities in the US, the study was approved both by the University of New Mexico Health Sciences Center Human Research Review Committee (HRRC# 22-016) and by the local tribal council. It included a memorandum of understanding outlining publications’ conditions. Following this memorandum, research updates, including the manuscript, has been shared with Tribal leaders and stakeholders and received their approval.

**Recruitment.** Following existing practices in other studies in the community, community health representatives posted and distributed flyers inviting participants. Focus groups followed all COVID-19-related guidelines that were in place in the community at the time of the research (2022). A meal was served with the focus group, as is customary. Transportation from home to the focus group was provided for any participants who needed it.

**Data collection and management.** No identifiers were collected. The participants were identified by a number and asked to refer to other group members by numbers. If identifiable information was accidentally shared in the discussion, the transcribers removed it from the transcriptions. The participants received $50 as an appreciation for sharing their time and expertise.

**Focus groups**. The team conducted four focus groups with older adults (4–7 in each group). Out of respect for Zuni guidelines, no inclusion or exclusion criteria were applied. Focus groups were selected as they are culturally congruent and effectively elicit the experiences of marginalized populations [25]. Members of the team have successfully conducted focus groups in diverse NM communities^,^ (including this Pueblo) on diverse topics, such as healthcare inequities [26] and health-related perceptions [27]. To ensure cultural appropriateness, two of the authors (DMG and MQ), are community members and healthcare workers. They led both the focus groups and transcriptions, which included expressions and narratives in their language, Shiwi. The focus groups took place at the Pueblo Health Initiative office. Additional moderators were also coauthors (E.L.P.; S.G.R.; J.L.P.; J.A.K.; G.A.R.). The questions focused on related topics, such as perceptions of aging (“What do think about when you hear the word aging?) and etiology (“what do you think causes memory loss?”). The moderators followed up the questions with each participant (e.g., “how about you, number four?”). The guide was semi-structured and allowed the moderators to follow up on topics raised, and to use probes and clarifying questions. The groups were recorded and transcribed by the Zuni moderators, resulting in 71 double-spaced pages and about 24,000 words.

**Thematic Analysis.** The analytic approach was inductive and interpretive. Using thematic analysis, the team sought to allow themes to emerge from participants’ narratives rather than impose an external framework. Thematic analysis was appropriate because it is well-suited to elucidating a group’s conceptualization of a phenomenon and to producing a detailed, nuanced account of patterns within the data [28]. Analysis was also informed by the constant comparative method, which supported ongoing comparison across participants’ accounts to identify commonalities, divergences, and emerging conceptual categories [29,30,31].

Coding proceeded through open, axial, and selective stages, without use of designated software to allow access to all team members. The first author conducted close line-by-line reading of transcripts, attending not only to what participants said but also to emotional tone and collective meaning-making reflected in agreement, silence, laughter, and shared stories. Initial open codes were refined through constant comparison and memo writing, with attention to recurrence, repetition, and forcefulness [28]. Codes were then organized into categories and subcategories during axial coding and collapsed into broader themes during selective coding [28]. Themes were retained when they reflected shared and patterned experiences across at least two focus groups; all final themes were ultimately expressed across all four groups.

Thematic analysis method [28] is an approach that is considered as “best suited to elucidating the specific nature of a given group’s conceptualization of the phenomenon under study” (p. 215). Our goal was to center the voices of participants by allowing their sensemaking of dementia and aging experiences to emerge. Inductive methods were used to learn about their construct and experiences of dementia and aging, consistent with the constant comparative method [29]. By focusing on these experiences, we aimed to “provide a more detailed and nuanced account […] of themes, within the data.” [30] (p. 11).

The first author read the transcripts line by line and identified instances in which participants discussed experiences and perceptions of aging and dementia. The words of those who shared these narratives were carefully examined to elucidate the emotional impact on participants. Similarly, reactions by other participants who reacted to them in agreement, laughter, silence, or sharing similar stories were noted. During open coding, the first author created a preliminary set of codes that were refined using the constant comparison of data including attention to commonalities and divergences. Themes were written and memos about their properties were created based on recurrence, repetition, and forcefulness [31]. In the following axial coding stage [29], themes were grouped according to their relationship and properties. The first author identified and grouped the initial codes into categories and subcategories that constituted meaningful clusters. These categories were refined and collapsed them into themes in selective coding by focusing on how the categories reflected the shared and patterned experiences of participants [32]. Themes were included if they were shared across at least two different focus groups. Overall, the team noted that the participants demonstrated good agreement. Not only did participants not express any explicit disagreement, but on most topics they echoed each other to indicate and create shared understanding. All the themes were expressed in all the focus groups.

Member checking [33] followed established protocol with the Pueblo. They included feedback from the community coauthors who participated in meetings during the analysis and following its conclusion. They concurred with the themes and emphasized the importance of special dementia care for participants. In addition, the Tribal leadership approved two drafts of this work, including this final one.

## 3. Results

Twenty participants joined our focus groups, including 14 (70%) women and six (30%) men. They ranged in age from 50 to 77 years, with a mean age of 64.3 years. Following the memorandum with the community and due to its small community size, these demographics are provided in aggregate form only to maintain anonymity. Six major themes emerged in our analysis, including (1) aging as a fear-evoking, communal experience, (2) perceptions of dementia as part of aging and proposed causes, (3) expressed interest in dementia-related information and services while acknowledging tradition, (4) barriers to specialized dementia care access, (5) resilience and Active Coping Strategies, (6) caregiving, strength in culture and traditions, and challenges, and (7) pandemic impact on aging in the community (see Table 1 for summary). Each quote includes the number of the focus group and the number of the participant. For example, the first participant in the second focus group is 2.1.

## 4. Overarching Themes

### 4.1. Aging as a Fear-Evoking, Communal Experience

[I am] worrying about other family members: grandkids and maybe somebody that’s your brother or sister that’s in the same boat as you are, but more severe than you are, you know? **And the worlds that bring us to depression […] When the worlds change and when we get old** [Participant 3.5, emphasis added].

As the above quote indicates, participants described aging as a communal, cross-generational, and cyclical experience that evoked fear and worry and involved changes to participants’ identity. Aging took place as they transitioned from young to old people and from caregivers to those needing care. Aging was not merely an individual process; it was also reflected by looking at their peers who have grown old with them. This experience of aging with others in the community elicited worries and fears for aging individuals themselves, for similar others, for their families, and for the community. One participant said:

“It’s scary […] I look at my friends here and we are all white-headed now, and it didn’t seem that long ago when we were still young and dancing.”

(1.1)

This participant also shared a concern about the impact of dementia on the families: “*I hope that the day won’t come where I won’t recognize my own family because I think that would really hurt them.*” (1.1).

The participants who expressed worries about memory loss, also raised fears and concerns about their ability to safely execute daily chores due to declining physical health, which often led to a loss of independence. A participant explained, “*I’m afraid to hold hot stuff just to put it on the table so I have to ask somebody to do that* [4.1].” For some, such experience of threat to personal safety served as a cue that they should not live alone. Similarly, participants identified the inability to take part in regular conversations as a signal of cognitive decline. They described how at times, other family members also noticed this decline and were prompted to seek help. For example, a participant shared that her daughter’s worries led her to take her to seek screening.

However, not all participants were concerned about memory loss. One participant described remembering negative events as a concern rather than concern related to aging:

I’m just the opposite; I can’t forget things. […] Sometimes it’s just irritating for me to still remember things that I don’t want to remember. […] I have trained myself to not really think about where I am stressing myself out. So, aging does not really concern me

[2.4]

These participants expressed the notion that forgetting can be a blissful escape from difficult memories.

### 4.2. Perceptions of Dementia as Part of Aging and Proposed Causes

At the beginning of the focus groups, when moderators asked about aging, participants typically responded by describing memory loss. They expressed both fear and humor in describing instances of memory loss. They often mentioned memory loss as an unavoidable part of aging, and did not assign different severity levels to different types of memory loss. For instance, one participant mentioned misplacing their glasses, a rather common “senior moment,” in the same breath as becoming disoriented while driving:

I’ll say where’s my glasses? […] and it would be on top of my head […]! I guess once you get old you know your mind is going buzzard […] Sometimes even though if I’m driving I got to see if I’m awake or what

(1.4)

Whereas participants did not offer a framework that distinguished between mild, “senior moments” to more severe red flags, when the healthcare providers/moderators shared such a framework, providing examples that distinguished between normal aging-related memory loss and dementia, this definition seemed to resonate with participants. They responded with stories about family and community members who became disoriented, and even potential experiences of delirium, as opposed to the initial stories of momentary forgetfulness. For instance, a participant noted how older community members used to think that they were participating in Pueblo cultural/religious ceremonies in the village’s plaza (old square).

They did what they used to do in the past. Like in the middle of the night they would go out in the plaza and sit out in the plaza while the family members are sleeping. And the neighbors would see and go knock on their door, “Your grandma or moms outside again, they’re thinking they’re dancing out there again.” Yeah. It’s kinda scary […]. They need more caring to be protected

[2.2]

The above description also provided the sense of community coping, where neighbors alerted family caregivers to the state of their loved ones. Moreover, it reveals the importance of cultural and religious traditions in the lives of the community and elders. Dementia can rob the community of the important participation of elders in these rituals. Therefore, the fear that community members mentioned, related not only to people who experienced dementia and their families, but to the whole community.

When the facilitators asked participants what they thought caused dementia, they seemed uncertain and provided a few possible explanations. While some used medical models, such as brain cell decay, diabetes, or vitamin deficiency, they seemed to resonate predominantly with psychosocial explanations, such as stress from social isolation and worry or trauma. Some assumed that people choose to forget negative experiences. For instance, when probed if medical conditions affected memory loss, the following conversation ensued:

Maybe Diabetes or let me see… what else I can think of… I don’t know, there’s a lot of other stuff that can add up to all that or maybe something happened to them a while back. […] most people think they just want to forget somehow? Yeah! […] poor diet. Being vitamin deficient!

[2.5]

Others in this group explained that stress, trauma and isolation caused memory loss and related these to societal changes in their community and loss of traditional relationships:

there’s a lot of memory loss because of the stress especially, with family members, family members don’t respect them […] and they just build [stress] inside themselves. “Nobody cares, so why should I remember that”? And they just start slowly forgetting about it. They lose it!

[2.4]

Another belief that participants shared was that dementia was unavoidable. A participant explained: “I don’t think there’s any other way trying to avoid dementia. I don’t think… it just naturally comes if you’re getting older, I guess. Is there a way for dementia to be avoided?” [4.5]. This participant’s ending his statement with a question to the team also indicated that many participants were not sure about dementia, and sought information from the team.

### 4.3. Expressed Interest in Dementia-Related Information and Services While Acknowledging Tradition

The question raised in the quote above (“Is there a way for dementia to be avoided?”)

was directed at the healthcare providers who participated in facilitating the focus group. The participant posed in the same breath as suggesting that dementia “*just naturally comes if you’re getting older, I guess.*” This, and similar instances, revealed that participants often questioned their own knowledge, and sought medical information when it was accessible to them. They were open to learning about dementia from the medical team, although they explained that talking about dementia was not traditionally discussed in the community, and that stigma and shame were involved at the present and might hinder current conversations. A participant [3.1] shared: “*our parents never mentioned anything about dementia or anything like that to us so we never knew what that meant […] I don’t think the community’s really open about that?* […] *they’re ashamed to talk about what’s ailing them.*” Another explained: “*In other words our parents never mentioned anything about dementia or anything like that to us so we never knew what that meant*” (4.6).

Along with acknowledgment of shame and stigma, participants felt that it was important to resist this stigma, and to talk about dementia. They explicitly and implicitly requested information. One person [3.4] described tensions between a desire for help and feeling helpless and ashamed without knowledge: “*They don’t want outsiders knowing what’s going on but yet, there’s that cry for help and you don’t know what to do.*” Whereas participants shared a strong desire to receive information about dementia, they did not necessarily articulate specific topics of the information they desired. One participant noted: “*We all just want to know more about this aging.*” Others specified that they hoped for more information from physicians, researchers and through pamphlets. One of them explained:

Trainings like this [are needed] because some people might not know what dementia is or what kind of things that they can do […] because I think brain can trigger aging symptoms.

[4.1]

Participants expressed enthusiasm about the opportunity to have open discussions: “*This is the first start, this is the first step! They never had a discussion like this about it! […] Thank you for bringing this up.*” [1.1]. These Focus groups seemed to offer participants a chance not only to reflect, but to ask providers questions about dementia and make sense of their experiences. A participant who had cared for a relative with dementia, but could not cope with his violent outbursts questioned if she could have kept caring for him at home with appropriate medical care: “*they never gave him medicine or anything, they just left him alone, but right now do they have stuff for them to control themselves?*” (1.4). Another participant in the group followed up with an inquiry about the etiology of dementia, early symptoms, and diagnosis: “*when you have those two diseases [dementia/AD], what’s the system of that? you’re getting it or is it in your system now or what age do you get that? […] How can you tell yourself if you’re getting it?*” (1.5). A different person asked: “*How would you spot those signs like I was saying […] ‘Oh you forgot something’ whether you were significant forgetting or not knowing?*” (3.4) and another echoed: “*Like early stages, or pre-stages.*” (3.3). These questions indicated both willingness to talk about dementia, and information prevention, early signs, diagnosis, and treatment information needs.

For example, one participant thanked the team:

Well first of all um, thank you for sharing on such an amazing topic you know because it’s amazing because I’m sure many of the, the stories that we tell will be the same […] The more we, we hear about each of our stories I think we can find the similarities and find that there is not much support for, for people who have Alzheimer’s and Dementia.

(2.1)

The participant also expressed a need for a brochure as an intervention. “I’m sure as this program goes along there will be some handouts regarding all of that and I guess we just kinda study up on our own”

### 4.4. Barriers to Specialized Dementia Care Access

Participants’ information needs as described above were consistent with their interest in specialized dementia services in their community. Some felt that there were no services available at all, not even for screening: “*But there’s no place to go [in the community]. You should take him up to this certain place you know so they can actually do a test*.” (2.5). Additionally, they described high provider turnover at the village clinic, and a lack of specialized training for healthcare providers on dementia as two interrelated barriers to dementia care. A participant described: “*before you know it, at your next appointment there’s a new doctor […] and they don’t really know themselves how to address issues of Alzheimer’s or Dementia.*”(2.1). This person, who was a caregiver to her mother, explained how lack of specialized care intensified barriers to accessing services due to providers’ lack of knowledge:

The first thing that you got to have [to access specialized services] is a doctor’s statement regarding the person’s condition […] if the doctor themselves don’t know if it’s dementia or Alzheimer’s or if it something else […] you can’t get any more help.

### 4.5. Resilience and Active Coping Strategies

As participants discussed their coping experiences and strategies in view of aging-related challenges and limited access to services, they shared notions of resilience, including the belief that one needs to keep on and continue to actively cope in the face of losses inherent to aging: “*We go through relatives that passed away but we just have to keep on going.*” (3.2). Participants also voiced the importance of active coping as a means of remaining healthy by trying to keep active through daily chores and exercise, as well as awareness and strategies to remember. During the third focus group, the following dialogue took place:

Participant #1. Everyday! I try to be active […] I think cooking is better.

Participant #5. Pressing the dough

Participant #6. All day work […] chopping wood and bring the woods in

However, they also brought up challenges:

Participant #6. I’m bad at chopping wood; I always fall.

As #6’s comment illustrates, while participants strove to be physically active, they often experienced barriers. Trying to exercise was described as a goal that was made difficult because of ailments, including the impact of long COVID-19 and a lack of opportunities in the community during the pandemic:

I like exercising or walking just maybe a short distance. […] when you start it’s like just go as much as you can and just motivate yourself, Okay do better tomorrow and I can do better the next day and all that. “Laughs”

(4.5)

The above quote echoed the importance of having a positive attitude and depicted the use of different coping strategies as key to coping with a multitude of challenges in her life. This participant demonstrated coping on the intrapersonal level by being positive and active, enjoying her family support by spending time with her daughter, and by contacting the father and the housing agency to get help with her haunted house.

Participants often referred to active coping by being aware of and focusing on their memory as “training the mind,” which they believed would avert memory loss, and allow them to cope:

I myself have to realize what I was doing, forgetting stuff, and so I started training my mind to have little things that could remind me or just um, let myself know where certain items are.

(2.4)

Resilience and coping strategies included accessing services, such as physical therapy, weight room, screening and well-being medical appointments. The narratives shared also included negative experiences in the medical system, leading to some participants’ mistrust, and reliance on self-advocacy. One participant said: “*I don’t tell nothing to the doctor’s cause I tried, I tried for help. They’ll give me […] an answer that they always give me ‘nothing’s wrong with you.’*” (1.5). This participant avoided seeking services at the clinic following a bad experience, when she had a concussion and was ignored by the provider. She also expressed doubts about the effectiveness of medications dispersed at the clinic and the ease at which they were prescribed in her community:

If I get sick-, I get sick. I don’t wanna go down there [the clinic]. Like I always say I ain’t their guinea pig! […] I look at my community, they’re all getting sick of this and that, too much medication given.

(1.5)

In contrast, participants shared that having positive aging role models in the family and the community facilitated coping: “*our great great grandma was like that she was really active and doing all these things around the house and she was 103.*” (3.5). Others described another aging family member that also engaged in traditional jewelry making: “*she’s always doing things, she’s still making jewelry and outside [activities] too.*” (2.2). Participants were trying to identify healthy aging behaviors based on these role models:

I swear it’s the coffee “Laughs” strong coffee because my mother was 74 years old or 75 yeah she drank coffee every day, and her memory was good; […] and she always ate healthy… she passed away due to COVID-19.

(3.7)

### 4.6. Caregiving, Strength in Culture and Traditions, and Challenges

The participants shared caregiving experiences, which they viewed as a cultural tradition that provided continuity and a way to honor older adults for their lifetime contributions to the community. Furthermore, caregiving was also perceived as a way to maintain culture, community, and cultural teaching. One participant explained *I really respect the things my grandmothers have taught me over the years so if I can help keep them* [older adults] *healthy, then they can continue to teach me things that I need to know* (3.1).

Caretakers described drawing strength from caregiving. Some participants indicated that caregiving provided them with a roadmap for their own aging. One participant shared:

I used to take care of older people: my aunts and relatives like that so I can easily tell I’m getting […] dementia. Hopefully, it’s not that but, I’m going to undergo an MRI next week.

(2.2)

At the same time, the participants noted that caregiving took a toll on caregivers’ health and on other roles and responsibilities they held. Caregivers often had to cope with personal health conditions, which impeded the care they were able to provide their loved ones with dementia. One person felt that her sister failed to care adequately for their mother owing to her own health challenges:

it’s like taking care of a baby 24/7 […] we didn’t know she was really neglected […] my sister, […] She herself needs caretaking too, because she goes to dialysis. I suggested […] home health and there’s other providers and resources to try and help her get a caregiver. [there is] so much a family can do

(2.3)

Other participants shared similar experiences of caring for relatives who had to be placed in nursing homes due to the severity of their conditions, and lack of local resources.

Some participants felt that there was a generational shift in the ability of families in the community to care for older adults. They reported community changes in how aging is manifested as well as in caregiving roles.

To this day, the parent is like taking care of themselves instead of the kids taking care of you, you know. It’s a hardship with the kids […] and as our grandparent passed away it’s really different […] Even though they were aging, they cooked, they cleaned, […] Now to this day […] single parents are doing their own way of doing everything for everybody in the household or with the friends who ever you see you help them out.

(1.5)

The above quote also underscores the importance that participants attributed to the responsibility of the community in caring for older adults who need help. They felt that in the past, older adults were active, and at the same time supported by their children. At the present, they described that many aging community members cannot rely on their children to care for them, which increased the significance of helping these older community members.

### 4.7. Pandemic Impact on Aging in the Community

We did not originally aim to examine the impact of the pandemic on the community, and therefore, we did not pose specific questions about it. However, at the time of data collection, participants brought up painful stories and indicated the devastating impacts of the pandemic on their community and lives. Whereas aging was experienced as a community, the pandemic not only led to loss of loved ones and participants’ health, but also interfered with the communal experience by severing ties. Some participants lost family members, and some were still recovering from or experiencing the impact of long-term COVID-19. Deteriorating health due to COVID-19 infection had a toll on social networks of participants and their health.

*I used to walk around the lake or whatever with the grandkids […] now I have a cane and I can’t go that far and I have my eyes doesn’t work on my left side*

(3.4)

In addition to personal losses and impacts on individuals’ health, participants described how the community faced reduced opportunities to socialize and to be active. A participant talked about the impact of closing the weight training room: “*It is hard for other people […] that used to go early in the morning like at 5 or 6 o’clock [to exercise] since with the COVID-19 it’s like they schedule their own like exercising marker.*” (2.2). In response, another participant noted how policies that eliminated social gatherings hurt older adults:

They used to do bake sales for their journey trips to other pueblos to visit their senior centers and they’ll share ideas of what to do […] some were so active, [they] went to the Senior Olympics. And that’s one thing that was really getting them together was the bake sales […] just being there watching and meeting other seniors.

(2.5)

This experience shows how the pandemic and isolation hurt social networks by eliminating important social activities between different local Indigenous communities and impacting their well-being and opportunities to be physically and socially active.

## 5. Discussion

Zuni participants’ perspectives on aging and dementia reflected a holistic, community-centered worldview in which physical, emotional, spiritual, and communal well-being are interconnected. Dementia-related decision-making was described as done at the family level, with strong kinship systems and respect for elders guiding caregiving practices. Dementia was understood as part of aging or as an imbalance influenced by stress, trauma, or social disruption. Participants described both traditional and Western approaches to care as often used together. They sought trust, relationships, and continuity with providers as critical for engagement. However, they were often unable to access either culturally centered or dementia-specialized care. They considered the impact of aging, and specifically of dementia, on their families and the whole community. Taken together, the themes suggest that aging and dementia were experienced as communal and emotionally charged processes, shaped by cultural and social beliefs, constrained by limited healthcare access and cultural disruption, and managed through family caregiving, resilience, and cultural traditions.

These perspectives were dialectical. While they often described memory loss as part of aging, they also wanted more medical information about dementia, including information about prevention, screening, diagnosis, treatment, and caregiving. While they described an ideal image of aging centered on resilience, activity, well-being, and cultural continuity, they also described the challenges, losses, and fears associated with aging. This dialectic is important because it challenges the assumption that viewing dementia as part of aging necessarily reflects disinterest in biomedical care, or serves as a barrier to care [2,5,34]. In this study, participants’ holistic understandings of dementia coexisted with a clear desire for specialized services and information.

Participants described being blocked from seeking care by a lack of access and expressed frustration at the level of care and lack of knowledge of providers at the local Indian Health Services (IHS) clinic, which did not specialize in dementia. This unmet need contributed to mistrust in Western medicine, illustrated by the participant who stated that she was “not a guinea pig.” However, this mistrust did not prevent many participants from seeking medical help, including specialized dementia screening and care.

Aging and caregiving experiences reflected both resilience and unmet needs. They described culture and tradition as sources of strength and as frameworks for understanding aging, caregiving, and responsibility to elders. At the same time, they described disruptions to these systems, including family members living outside the village, reduced opportunities for intergenerational interaction, and limited access to culturally centered and dementia-specialized care. These disruptions complicated caregiving, family communication, and the community’s ability to support older adults. Thus, participants’ experiences cannot be understood only as evidence of cultural strength or only as evidence of healthcare need; rather, they reveal how cultural strengths and structural barriers coexist. These findings align with studies in other Indigenous communities that describe aging as a communal process [1,6,8,14,35]. However, our findings extend this literature by showing that communal aging was not only a source of strength, but also a source of shared loss, sadness, and concern as participants witnessed the aging and decline of peers, family members, and the broader community.

Community-level factors were also evident in participants’ experiences of caregiving, as well as for aging that were grounded in their culture and traditions. They felt that their culture provided a responsibility and a framework for aging and for caregiving. However, such frameworks were often disrupted, both for their own coping aging and for caregiving. For example, having family members who lived outside of the village complicated both caregiving and family communication. Similarly, they described familial stressors, such as having single parent homes and consequent cultural losses as creating loss of frameworks on how to age well. They viewed such disruptions to culture as hurting both individuals’ well-being and the ability of the community to care for older adults.

Participants also shared that dementia is not widely discussed in the community and is often a hushed topic. Many felt that they did not know enough about dementia and were interested in learning more through in-person conversations. The focus groups themselves appeared to model one culturally appropriate format for dialogue, suggesting that community-based, relational, and discussion-oriented approaches may be useful for dementia education. Such approaches may be especially important when dementia is associated with fear, silence, shame, or uncertainty.

To our knowledge, our study is the first to report the impact of COVID-19 on this population in the context of aging. Indigenous communities in New Mexico endured a disproportionate impact, evident in morbidity and mortality [36]. The participants shared narratives and personal experiences about the loss of personal health, loved ones, as well as the impact of isolation and social functions and services that had direct effects on their health. Given the importance of communal coping, this social isolation was particularly devastating. It seems that in the midst of the pandemic, a very narrow perception of health and illness was implemented in fear of infection, resulting in the cessation of important gathering and socialization opportunities that, at the time of the focus groups, were no longer offered. The pandemic exacerbated existing challenges and posed a major disruption to healthy aging. It impacted their personal health both directly and indirectly through isolation and limited access to services and the community at large. Future research should examine not only the pandemic’s impacts on morbidity and mortality [37] but also the impact of public health strategies, and it should involve community members in designing aging-friendly strategies for future contingencies.

Rigor was supported through a community-engaged qualitative design that centered cultural responsiveness, relational trust, and participant-led meaning-making. The study was approved by both the University of New Mexico HRRC and Zuni Tribal leadership, followed a memorandum of understanding with the community, and involved community-member coauthors who co-led focus groups and transcription, including preservation of Siwi expressions and narratives. Analytic rigor was strengthened through inductive thematic analysis, constant comparison, memo writing, and open, axial, and selective coding, with themes retained only when they appeared across at least two focus groups and ultimately confirmed across all four. Trustworthiness was further enhanced through member checking with community coauthors and review and approval of manuscript drafts by Tribal leadership.

This study was not without limitations. First, whereas the team had community members as moderators, it was also the product of a new collaboration, and not all team members were familiar with the community, which might have also impacted what they were willing to share. Second, the focus group format may have discouraged disclosure of stigmatized or minority views about dementia, including shame, blame, fear, or disagreement with dominant group perspectives. Participants who held less common views may have deferred to group consensus or avoided sharing perspectives that could be perceived as disrespectful to elders, families, or community norms. Larger and longer-term studies are needed in collaboration with the community, including studies that use multiple methods such as individual interviews, family interviews, listening circles, and community forums.

Our findings support the importance of dementia-specialized outreach in this community, paired with culturally centered and trust-building approaches. Future practice should move beyond general calls for education and services toward concrete, community-designed strategies. One actionable recommendation is to develop a culturally adapted dementia screening and referral protocol co-designed with Zuni community health workers, older adults, caregivers, clinicians, and tribal health leadership. Such a protocol could include plain-language explanations of dementia, guidance for family-centered conversations, culturally appropriate screening procedures, referral pathways for specialized care, and follow-up support for caregivers. Because provider turnover is high in the community, dementia-related training should be ongoing. Training should include culturally centered communication, dementia diagnosis and care, family caregiving dynamics, and strategies for discussing dementia in ways that reduce stigma while preserving diagnostic clarity.

The importance of culturally appropriate interventions should not diminish from the importance of dementia-specialized care services in this community, which goes hand in hand with the need for culturally centered interventions to meet the needs of this community for information, specialized care, and support for people with dementia and their families. Moreover, given the strong negative emotions of fear and worry that participants described, such future culture-centered interventions should address emotional response to aging and to dementia, including fear and a sense of loss on the communal level. Healthcare providers and caregivers in the community should receive training in dementia care, including culture-centered information. Due to the high provider turnover in the community, such training should be continuous. Interventions should also consider connections with community older adults and caregivers who live outside of the village and maintain consistent communication and services to these individuals, who are an inherent part of the community despite their geographic distance.

## Figures and Tables

**Table 1:** Overarching themes.

Theme	Summary
1. Aging as a fear-evoking, communal experience	Participants described aging as something experienced both personally and collectively, as they watched themselves and others grow older together. This process brought fear on the personal level due to memory loss, declining abilities, loss of independence, and changing roles and identity, including moving from being a caregiver to needing care. It also brought fear due to community loss.
2. Perceptions of dementia as part of aging and proposed causes	Many participants saw memory loss and dementia as an unavoidable part of aging and did not always distinguish between ordinary forgetfulness and more serious impairment. When moderators explained dementia in more detail, participants connected it to more severe experiences they had seen in relatives and community members. They suggested possible causes that included medical explanations such as diabetes or vitamin deficiency, stress, trauma, isolation, and social/cultural changes.
3. Expressed interest in dementia-related information and services while acknowledging tradition	Participants showed strong interest in learning more about dementia. They explained that dementia was not openly discussed in the community in the past and that stigma could make these conversations difficult. Even so, they welcomed the focus groups as a rare and important chance to ask questions, talk openly, and seek medical guidance.
4. Barriers to specialized dementia care access	Participants described major obstacles to getting dementia-related care in their community. They reported few or no local services, frequent provider turnover, and limited provider expertise in dementia care. These problems made it hard for families to access care.
5. Resilience and Active Coping Strategies	Participants emphasized the need to keep going despite aging-related losses and limitations. They described coping by staying active through chores and exercise, using reminders and “training the mind,” maintaining a positive outlook, relying on family support, and looking to older relatives as role models. Some highlighted advocating for themselves when medical care felt inadequate, acknowledging mistrust and negative healthcare experiences.
6. Caregiving, strength in culture and traditions, and challenges	Caregiving was viewed as a meaningful cultural responsibility that honors elders, preserves tradition, and allows older adults to continue teaching younger generations. At the same time, caregiving was described as demanding and stressful, especially when caregivers had their own health problems or when dementia symptoms became more severe. Limited local services made caregiving even harder, and some participants felt that generational changes were weakening families’ ability to care for elders in traditional ways.
7. Pandemic impact on aging in the community	COVID-19 had a major impact on participants’ health, mobility, social lives, and well-being. Participants shared long COVID symptoms, reduced strength, brain fog, loss of independence, family loss and ongoing grief. The pandemic also disrupted gatherings and senior activities, weakening community and intertribal social connections that had supported healthy aging.

## Data Availability

De-identified qualitative data may be made available upon reasonable request and with approval from the Zuni Pueblo Tribal Council and the corresponding author.
